# Formation of nanostructured metallic glass thin films upon sputtering

**DOI:** 10.1016/j.heliyon.2016.e00228

**Published:** 2017-01-09

**Authors:** Sergey V. Ketov, Rastko Joksimovic, Guoqiang Xie, Artem Trifonov, Kazue Kurihara, Dmitri V. Louzguine-Luzgin

**Affiliations:** a WPI Advanced Institute for Materials Research, Tohoku University, Aoba-Ku, Sendai 980-8577, Japan; b Institute for Materials Research, Tohoku University, Aoba-Ku, Sendai 980-8577, Japan; c Skobeltsyn Institute of Nuclear Physics, Lomonosov Moscow State University, 1(2), Leninskie Gory, GSP-1, Moscow 119991, Russia; d Physics Faculty, Lomonosov Moscow State University, 119991 Moscow 119991, Russia; e National University of Science and Technology “MISiS”, Moscow, 119049, Russia

**Keywords:** Engineering, Materials science, Nanotechnology

## Abstract

Morphology evolution of the multicomponent metallic glass film obtained by radio frequency (RF) magnetron sputtering was investigated in the present work. Two modes of metallic glass sputtering were distinguished: smooth film mode and clustered film mode. The sputtering parameters, which have the most influence on the sputtering modes, were determined. As a result, amorphous Ni-Nb thin films with a smooth surface and nanoglassy structure were deposited on silica float glass and Si substrates. The phase composition of the target appeared to have a significant influence on the chemical composition of the deposited amorphous thin film. The differences in charge transport and nanomechanical properties between the smooth and nanoglassy Ni-Nb film were also determined.

## 1 Introduction

Modern nanotechnology is unimaginable without thin films. Inorganic thin films with highly ordered crystal structure have already been used for a long time. Moreover, amorphous thin films found a huge interest recently with the development of organic, flexible and transparent electronics [1, 2, 3]. The lack of the long-range order gives glassy thin films unique properties such as high mechanical strength, hardness, corrosion resistance, wear resistance *etc.* These become useful for amorphous film applications in microelectromechanical MEMS devices, microelectronics, biochemical and others [4, 5, 6, 7]. The most widely used methods for production of the amorphous thin films are physical vapor deposition techniques, one of which is magnetron sputtering. This method is capable of production both ultra smooth films and crystalline nanoclusters [8, 9, 10, 11].

Nanostructured metallic glasses were obtained in the end of 20th century by the compaction of nanoparticles made by inert gas condensation. They were found to have some unusual properties such as emergence of interfacial ferromagnetism in consolidated Fe_90_Sc_10_ nanoglass [12]. Recently Au-, Pd-, Ti-and Zr-based metallic nanoglasses, consisting of spherical particles separated by metallic glassy interfaces, were produced from the powder targets by the magnetron sputtering method [13, 14, 15, 16]. These films appeared to form hierarchical and fractal structures which promotes high biocompatibility and catalytic activity among other enhanced properties [13, 15, 16].

The film structure evolution upon sputtering and physical aspects of sputtering technology was considerably investigated since the end of 20th century [17, 18, 19, 20, 21]. However, most of the works were done on the crystalline films sputtered from a single pure element target. The lack of long range order in amorphous films might result in much different film structure and morphology evolution. The processes that lead to the formation of nanoglasses are still not well understood. In the present work, we investigate an influence of the sputtering parameters such as gas pressure, sputtering power etc. on the structure and properties of amorphous Ni-Nb films.

## 2 Materials and methods

**Fig. 1:**
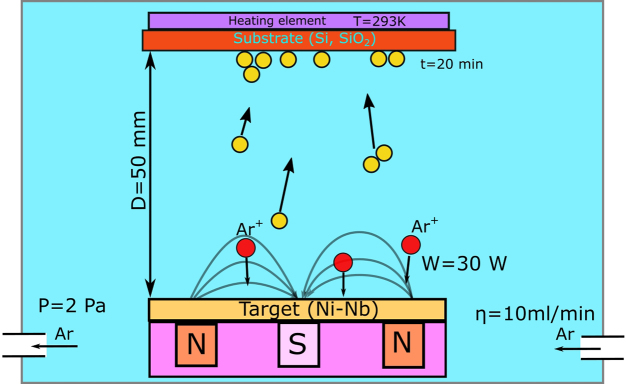
Schematic view of the experimental setup. The initial sputtering conditions were: W = 30 W, D = 50 mm, P = 2 Pa, η = 10 ml/min, T = 293 K, t = 20 min where W - discharge power, D distance between the target and the substrate, P - argon pressure inside the chamber during the sputtering, η - argon flow rate, t - deposition time, T - substrate temperature.

Ni_62_Nb_38_ target of 50 mm in diameter and 2 mm in thickness for sputtering machine was cut from a master alloy, prepared by arc melting of the mixture of pure elements (99.9% purity). This glassy alloy has a good glass forming ability GFA [22, 23] and its atomic structure, crystallization [24] as well as surface oxidation behavior was carefully studied [25]. Thin films were prepared by magnetron radio frequency (RF) sputtering technique (TOEI SP-3003-33) with initial pressure inside the chamber of 10^−4^ Pa without any special cooling techniques, though in some experiments the substrate was deliberately heated up. The thickness of the films varied from 100 nm to 500 nm. The experimental setup scheme one can find in Fig. 1. The experimental parameters were varied in such a way that one parameter was changing while others stayed the same as for the initial conditions, if otherwise is not specified in the text. Si wafer with an oxidized surface layer and silica floating glass (Matsunami glass Ind., Ltd., Japan) (SFG) were used as substrates for sputtering. Before sputtering the substrates were kept for 30 min in piranha solution (75 vol.% H_2_SO_4_ + 25 vol.% H_2_O_2_(30%)) to clean the surface from the organic contaminates and enhance adhesion. The substrates then were successively cleaned with distilled purified water.

The structure, chemical and phase composition of the samples were examined by scanning electron microscopy (SEM Hitachi S-4800) and x-ray diffraction (XRD) with Cu Kα radiation (Rigaku Smart lab). Surface roughness was measured by atomic force microscopy (AFM). The samples were first cleaned with a pressurized nitrogen flow. AFM imaging was carried out with a NanoNavi instrument operating in contact mode. Height images were recorded using double triangle cantilever Si probes and a 20 μm scanner. A smaller cantilever (100 μm length and less than 10 nm tip radii) was used for high precision. The scanned area was 1 μm^2^ at the scan rate of 0.86 Hz. The root mean square (RMS) and peak to valley (P-V) indices were obtained by averaging the values obtained from three different AFM images over the sample.

Local current-voltage characteristics of Ni-Nb amorphous films were measured using scanning probe microscope AIST-NT SPM (model SmartSPM-1000) at ambient conditions. The experimental procedure is described elsewhere [26]. The same AFM machine was used for scratch test. Diamond single crystal cantilevers with a typical spring constant of 40 N/m were used for this experiment. As the spring constant is rather large and the Young’s modulus of the diamond tip is very high compared with the film the deformation of the tip was considered as negligible. Nanoindentation was performed using a Nano-Hardness Tester instrument. A Berkovich diamond indenter was used with the continuous stiffness measurement (CSM) method; it offers a direct measure of hardness, and elastic modulus during the loading portion of an indentation test. A three-sided pyramid diamond Berkovich indenter with the radius of 20 nm and normal angle of 65.3° between the tip axis and the faces of triangular pyramid was used. Step indentation matrix was about 20 micron and the number of points was 20.

## 3 Results and discussion

### 3.1 Film preparation and structure characterization

**Fig. 2:**
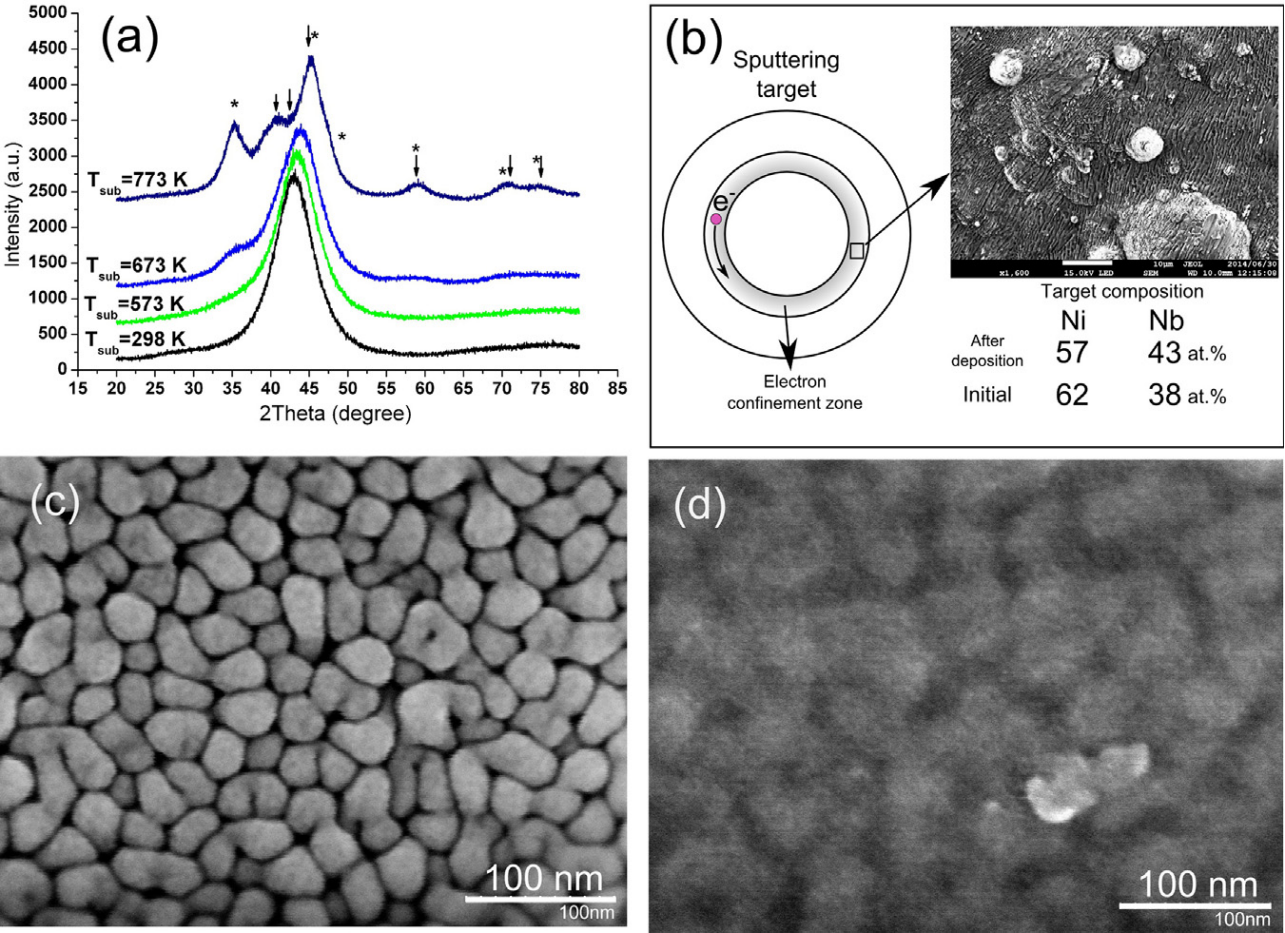
X-ray diffraction patterns of the thin films (star symbol is for SiO_2_ (Pearson symbol cP12) and arrow symbol is for Ni (hP2/1) reflexes) (a) and schematic view of the sputtering target from above (insert − SEM image of Ni-Nb target after several hours of sputtering) (b). SEM images of sputtered film in following conditions W = 120 W, P = 0.4 Pa, T = 673 K (c), T = 773 K (d).

X-ray diffraction analysis revealed that even though the sputtering was done on the substrate without cooling, the structure of thin films was amorphous (Fig. 2a).

The morphology of the thin film surface highly depends on the sputtering conditions and can change from a smooth to very rough state. In order to determine which of the sputtering parameters influence the structure of the film most of all the following regimes were chosen: discharge power (W = 20–300 W), distance between the target and the substrate (D = 50–100 mm), argon pressure inside the chamber during the sputtering (P = 0.4–10 Pa), argon flow rate (η = 5–20 ml/min), deposition time (t = 2–50 min), substrate temperature (T = 293–773 K). The initial standard sputtering conditions were: W = 30 W, D = 50 mm, P = 2 Pa, η = 10 ml/min, T = 293 K, t = 20 min. These parameters were set up as the initial state throughout all experiments unless otherwise specified.

**Fig. 3:**
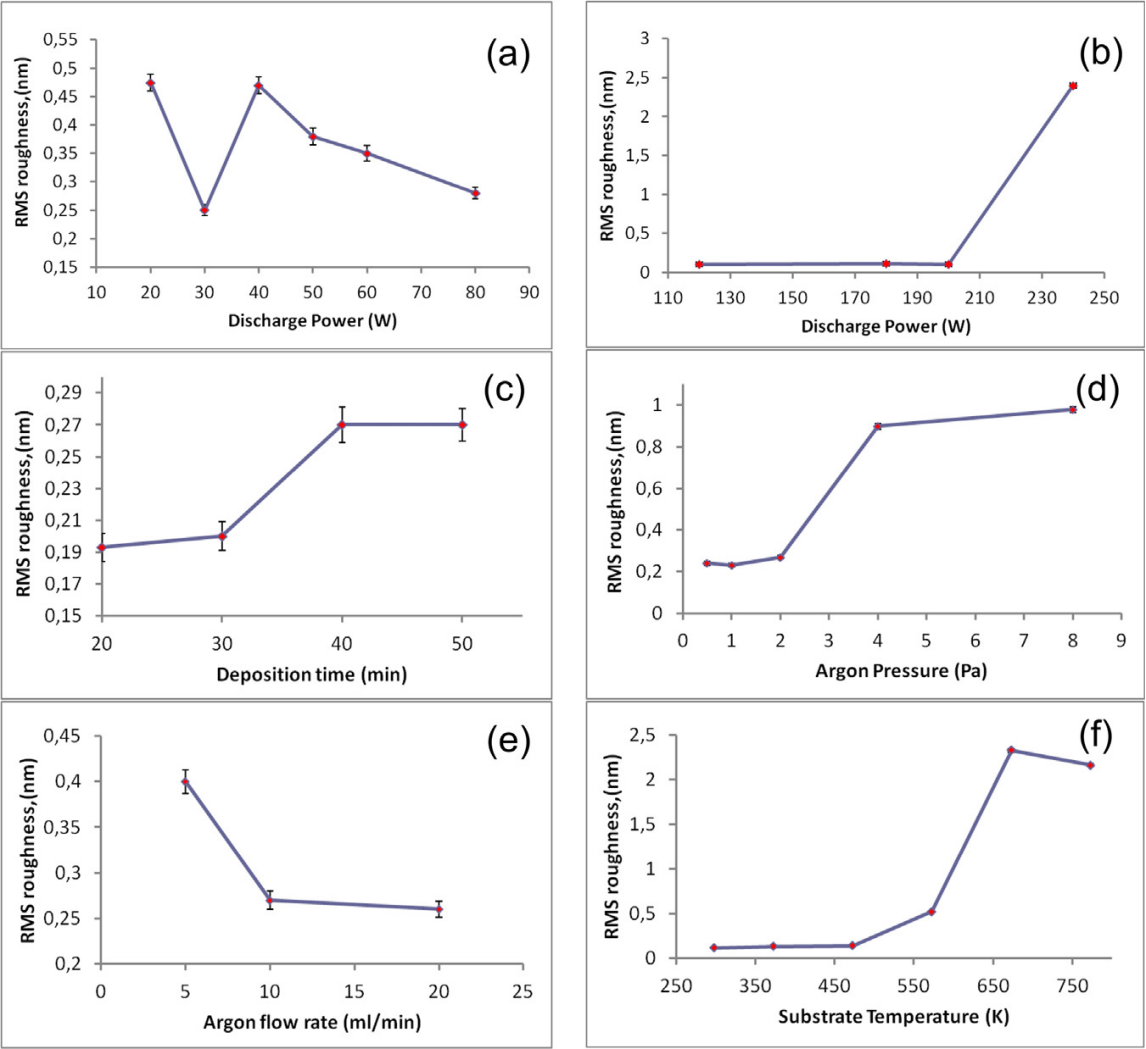
Dependence of the mean squared roughness of the Ni-Nb thin films from the following sputtering parameters: discharge power (substrate - SFG glass (a), silicon (b), t = 10 min, P = 0.4 Pa), deposition time (substrate SFG) (c), argon pressure (t = 50 min, substrate SFG) (d), argon flow rate (t = 50 min, P = 1 Pa - substrate SFG) (e), substrate temperature (W = 120 W, P = 0.4 Pa - substrate Si) (f). Lines here that connect the data points are just guidelines.

**Fig. 4:**
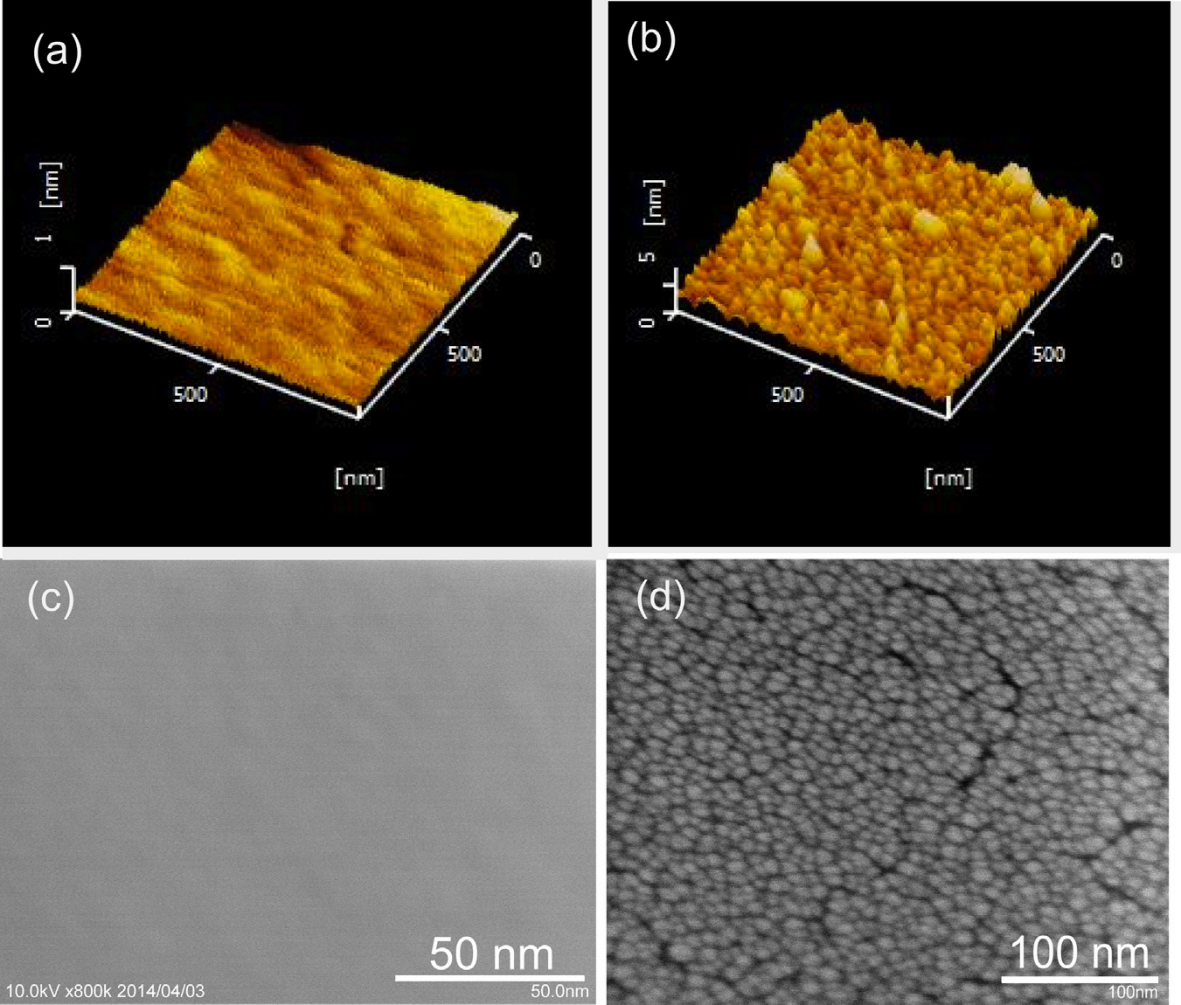
AFM (a, b) and SEM (c, d) images of the sputtered Ni-Nb thin films deposited under following conditions: Si substrate W = 120 W, P = 0.4 Pa, T = 293 K, η = 10 ml/min, t = 2 min and W = 100 W, P = 10 Pa, η = 5 ml/min, T = 293 K, t = 2 min respectively.

Following the sputtering parameters variation, the quality of the thin films surface was determined by AFM measurements. Variations of the root mean square (RMS) roughness parameter obtained for different sputtering regimes are presented in Fig. 3. The initial RMS of the substrates was 0.055 nm for Si wafer and 0.11 for silica float glass (SFG). AFM and SEM images of typical films with smooth surface and nanoglassy structure can be found in Fig. 4.

Sputtering differs from other physical vapor deposition techniques by involving intermediate gas between the target and substrate. AFM roughness measurements revealed that argon pressure and discharge power (Fig. 3a, b and d) have the strongest influence on the roughness of the film. Similar results were reported in the work [18] for crystalline materials. Thornton developed a zone classification of the film morphology evolution upon sputtering with different argon pressure and homologous substrate temperature (T/Tm, where T is the substrate temperature, Tm is the melting temperature of the target). This zone classification is purely empirical and do not explain the physical processes that lead to each kind of film structure. Three basic modes of the atomic film deposition were characterized: island or Volmer-Weber, layer plus island or Stranski-Krastanov, layer or Frank-van der Merwe growth modes [17]. However, these modes can be applied only to the equilibrium film growth (like for example in molecular beam epitaxy). Under unequilibrium sputtering conditions in the present experiments (not atomically flat substrate and interactions between the atoms from the target and intermediate argon) shadowing and re-emission processes start to play a big role in the film structure formation [8, 21, 27].

**Fig. 5:**
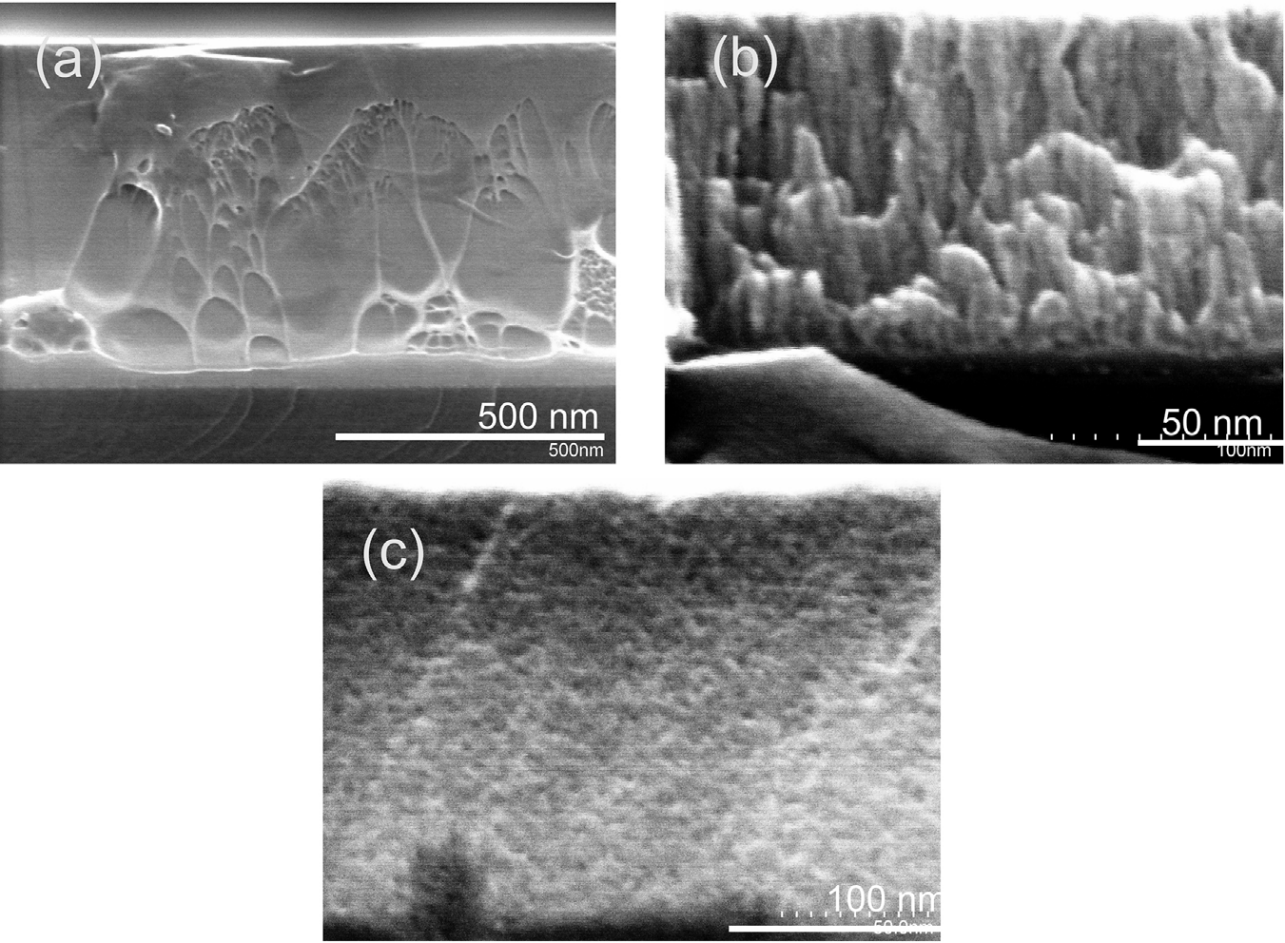
SEM cross sectional fracture images of thin films sputtered in atomic mode (a), mixed atomic-cluster deposition mode (b) and cluster growth mode (c).

Low argon pressure, high discharge power and small distance between the substrate and target decrease the chance of interaction of target atoms with argon and favor a smooth thin film formation (Fig. 3c, Fig. 4a, c). In the present work, the lowest argon pressure, at which sputtering plasma could be stabilized, was 0.4 Pa. Without substrate cooling and at high enough discharge power fast atoms from the target, having high kinetic energy can kick out atoms from unfavorable positions on the film surface, therefore making the film surface smoother (Fig. 3b W = 100–200 W). However, at a certain discharge power this process greatly intensifies and increases RMS (Fig. 3b, W = 240 W). A vein pattern can be seen in the SEM image of the fracture surface of the smooth thin film (Fig. 5a). It is typical for fracture surface of the cast bulk metallic glassy samples [28, 29]. In the present work, roughness of the thin film sputtered at the same conditions (W = 200 W) on silica float glass (relatively rough) and on Si substrate (smooth) was 0.235 nm and 0.1 nm respectively. Direct atomic deposition on the substrate with surface roughness of several interatomic distances can lead to the shadowing effect [27].

According to the works on clustering of pure elements upon sputtering [10, 30, 31], increase of the argon pressure and decrease of the discharge power decreases the mean free path of Ni and Nb atoms as their chances to interact with argon atoms increases. At some point, due to these interactions, initial impulse vectors of the atoms become chaotic and atoms start to interact between each other, forming atomic clusters. As shown in Ref [32] clustering of crystalline Ti already occurred at the distance 30 mm from the target at argon pressure 10 Pa. In the present work, the results were similar: amorphous clusters were already formed at 10 Pa and 50 mm distance (Fig. 4b, d).

At high argon pressure inside the chamber, the argon flow rate also begins to play a significant role in the clustering process. Heavy clusters with relatively slow motion speed can be sucked away from the chamber with the argon gas flux and, thus, high gas flow rate does not promote clustering (Fig. 3e). High gas flow rate also decreases the stability of discharge plasma at low argon pressures. In the present work high plasma stability at the gas flow rate 20 ml/min was achieved only at the gas pressure 0.6 Pa and above. Thus, glassy films with smooth surface were obtained at the gas flow rate of 10 ml/min.

We should note that two modes of clustering can be distinguished: cluster growth on the film surface (high argon pressure and relatively high discharge power) and cluster deposition (high argon pressure and low discharge power).

Cluster growth can lead to the columnar structure [18]. In the cluster growth mode a cross-sectional SEM image of the nanoglassy sample (Fig. 5b) has a columnar like fracture surface but the film still has multi clustered structure with large distribution of the cluster size [9, 14]. On the other hand, formation of the columns of the spheroid clusters is responsible for the formation of a fractal hierarchical structure on the surface and even surface cracks (owing to internal stresses) at the latter deposition stage [14]. Initial deposition conditions are in the cluster growth zone. Therefore, the surface of the film becomes rougher with deposition time (Fig. 3c). It is interesting, that amorphous clusters when contact the neighboring clusters upon growth retain the glassy boundaries, which have less densely packed structure [14]. In the work Ref. [33] to prevent the clustering effect caused by shadowing it was proposed to apply a negative potential to the substrate. Argon ions involved in the sputtering process consequently bombarded the substrate’s surface and smooths it out. In the present work, fast atoms from the target were used for the roughness improvement. As one can notice the RMS of the thin films decreases continually with the increase of the discharge power (Fig. 3a, b). Energy of the fast atoms is high enough to cause the reemission of the atoms from the unfavorable positions on the surface of the thin films.

Cluster deposition mode is still a complicated and not well-understood process [34]. Depending on the conditions and film thickness, deposition can be separated into several elementary processes like adsorption of a cluster by deposition, diffusion of the isolated clusters on the substrate, formation of an island of two monomers by contact of two monomers (nucleation), growth of a supported island by incorporation of a diffusing cluster; evaporation of an adsorbed cluster and island diffusion [34]. Each of them have its own characteristic time and can depend on each other. For example, surface roughness of the substrate play an important role as a perfect trap in the cluster deposition mode. Fracture of the thin films after cluster deposition is going along the borders of neighboring clusters (Fig. 5c) and appeared to be sponge like. The deposited clusters also can form hierarchical and fractal structures [34, 35, 36]. Having such kind of structures can benefit in catalytic activity and biomedical applications. Clustered glassy or crystallized Ni-Nb film can attract interest as magnetic quantum dots [25].

The substrate temperature T_sub_ at certain deposition conditions was found to play a big role in a cluster formation (Fig. 2a). In the present experiment the substrate was heated from 293 K up to 773 K. Increased mobility of the surface and bulk atoms at high substrate temperatures improved cluster-cluster coalescence and helped to form amorphous clusters of more than 50 nm in diameter (Fig. 2c) which increased porosity and roughness of the films (Fig. 3f). However, at a certain substrate temperature clusters begin to crystallize (Fig. 2a, d). This results in the formation of nanoparticles of metastable Ni (hP2/1) [37] and slight decrease of the surface roughness (Fig. 3f). Also at 773 K crystalline SiO_2_(cP12) phase was found on the XRD pattern. It possibly appears on the pattern from the parts of the substrate that were covered by the fixation screws during the deposition.

In the present work it appeared that the substrate temperature during deposition that leads to film crystallization is more than 100 degrees less than crystallization temperature of the film of the same composition but deposited on the cold substrate found in ref. [23] (T_c_ ≈ 700 K for Ni_68_Nb_32_). Formation of the crystalline nuclei during film deposition requires less energy because activation of surface diffusion is much easier than in bulk. We also should keep in mind that adatoms transfer their kinetic energy to the surface of the substrate upon the collision, which increases the energy of the film surface even more.

X-ray energy-dispersive analysis in SEM showed that chemical composition of the film did not match the composition of the target. It was found that the surface composition of the target as well as composition of the thin films changed after several hours of sputtering (Fig. 2b) from the initial Ni_62_Nb_38_ to Ni_57_Nb_43_ and Ni_68_Nb_32_, respectively. The composition presented for the film sputtered with initial conditions.

According to the phase diagram [38] Ni-Nb target with atomic composition of Ni_62_Nb_38_, have a eutectic structure of two crystalline phases Ni_3_Nb and Ni_6_Nb_7_, which also form on crystallization of the glass [24]. Having higher sputtering yield nickel (maximum sputtering yield S_max_ = 6 atoms per Ar^+^ ion) has more chances to leave the target than niobium (S_max_ = 1.87 atoms per Ar^+^ ion) [39, 40]. The presented sputtering yields were obtained for pure elements, but, to some extend, they can also be applied to the intermetallic compounds. Ni_3_Nb phase contains more Ni than Ni_6_Nb_7_ and would evaporate upon sputtering more quickly. This would shift the chemical composition of the film more towards higher Ni content which was observed in the present work (intended composition Ni_62_Nb_38_, while Ni_68_Nb_32_ after sputtering). Also after sputtering, the surface of the target found to have less Ni atoms than it was initially (Fig. 2b). It can be noticed from the SEM image (Fig. 2b) that degradation of the Ni-Nb target after several hours of sputtering occurred not homogeneously and resulted in formation of the surface with a mesh like structure. It appears that for such kind of target with eutectic structure even long presputtering will not equilibrate the average chemical composition. There always will be a shift towards the element with higher sputtering yield. Here, one also should not forget about the atomic weight difference of the elements and the fact that atoms leaving the target can be ionized and can return back to the target which also influences the final chemical composition of the film. We did not check the influence of sputtering parameters on chemical composition, however, it should mostly depend on the discharge power and distance between the target and substrate [9]. The chemical composition mismatch was also reported in Ref. [9] upon sputtering of the Zr-Cu-Ni-Al target. However, with increasing of the sputtering power the difference decreased. Therefore, from the prospective of multi component target sputtering with controllable thin film chemical composition, atoms in the target should have similar sputtering yields or spatial distribution of the atoms of different elements in the target should be homogeneous, like in solid solutions, single phase system or fully amorphous targets [9, 41].

### 3.2 Properties of the smooth and nanoglass films

**Fig. 6:**
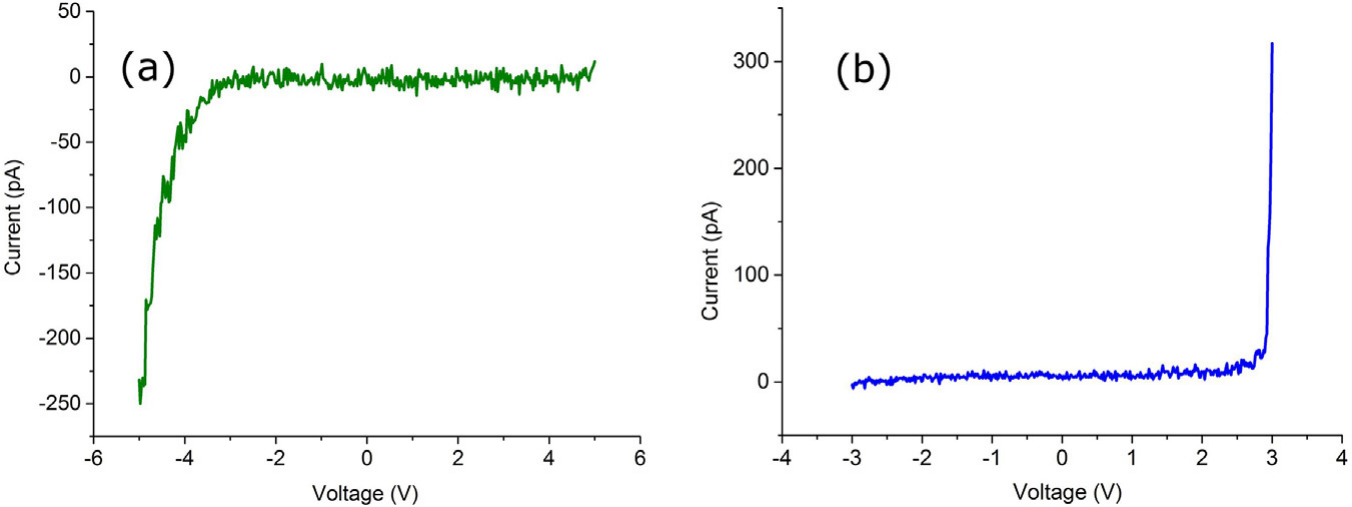
Current-voltage characteristics for nanoglass Ni-Nb film (applied load - 60 nN) a) as sputtered after natural oxidation in an ambient air, b) after oxidation in dry air at 573 K for 30 min.

Properties of the thin films are mostly governed by their composition and structure. However, if thickness of the films is of the nanometer order surface start to play a big role. Any film (except for the films made of noble metals only) tends to oxidize at an ambient air conditions. Thickness of the native surface oxide even for amorphous alloys, which have no any internal defects such as grain boundaries, could vary significantly from as small as 1 nm [42] and up to 30 nm in case of Cu-Zr-Al alloys [43]. For Ni-Nb glass it was found to be 3–4 nm [26, 44, 45]. Therefore, as it has been shown before, influence of the surface oxide to the thin film properties can be crucial. In case of Ni-Nb amorphous alloys, Nb forms an amorphous dilute oxide on the surface and Ni, as an element less attracted to the oxygen, diffuses deeper inside the sample [25]. Nb surface oxide influences the charge transport of the Ni-Nb film with the smooth surface [26]. Similar behavior is observed in the nanoglasses (Fig. 6). However, the passing current depends very much on the barrier thickness. For Ni-Nb nanoglass current is much larger than that observed for the smooth films, which means that the oxide thickness is smaller, therefore, tunnel junctions become suppressed. Also, the voltage at which conductance starts in the nanoglass is 3 V (Fig. 6), which is significantly lower than that of the smooth Ni-Nb glassy sample studied earlier (around 5 V) [26]. These changes are still unclear and need more investigation.

**Fig. 7:**
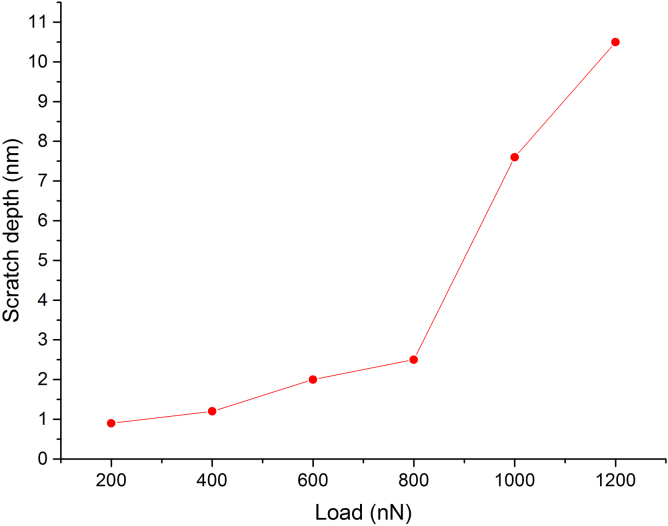
The results of the AFM scratch test on nanoglass Ni-Nb film. Line here that connects the data points is just a guideline.

The nanoscale scratch test of the nanograined glass film performed in AFM showed sudden change of the scratch depth near 2 nm (Fig. 7) which is attributed to the failure of the surface oxide. Similar behavior of the scratch depth against load was observed on other metallic glasses [46]. Nanograined glasses appeared to be in an ultrastable dense state [47] and diffusion of oxygen in them is hampered which results in smaller oxide thickness. However, nanoindentation measurements revealed that nano hardness of the smooth film is much higher than that of the nanoglass (16 GPa and 8,6 GPa respectively). This can be the result of the heterogeneous nature of the nanoglassy films. The existence of the boundaries with larger excess volume between the grains [14] makes these films more susceptible to the boundary deformation or fracture.

## 4 Conclusions

In conclusion, it is shown that RF magnetron sputtering is a method capable of preparation of amorphous thin film in two modes: smooth atomic and cluster modes and the preparation conditions were defined. Smooth (with the roughness around 0.1 nm) and nanoglass (with the cluster size ranging from 3 nm to more than 50 nm) amorphous thin films were obtained for the Ni-Nb system alloy. The discharge power and argon gas pressure have the strongest influence on the deposition modes. It was found that phase composition of the target can influence the chemical composition of the deposited thin film. Multi component targets should have homogeneous elemental spatial distribution for controllable chemical composition of the thin film.

Despite the structure differences, the smooth and nanoglass films exhibit similar conductance behavior. The thickness of native oxide layer on nanoglassy film appeared to be smaller because of its ultrastable nature. However, due to the grain structure of nanoglass its hardness is much smaller than that of the smooth film.

## Declarations

### Author contribution statement

Sergey Ketov: Conceived and designed the experiments; Performed the experiments; Analyzed and interpreted the data; contributed reagents, materials, analysis tools or data; Wrote the paper.

Rastko Joksimovic: Conceived and designed the experiments; Performed the experiments.

Guoqiang Xie: Analyzed and interpreted the data; contributed reagents, materials, analysis tools or data.

Artem Trifonov: Performed the experiments; Analyzed and interpreted the data.

Kazue Kurihara: Conceived and designed the experiments; Analyzed and interpreted the data; contributed reagents, materials, analysis tools or data.

Dmitri Louzguine-Luzgin: Conceived and designed the experiments; Analyzed and interpreted the data; contributed reagents, materials, analysis tools or data; wrote the paper.

### Funding statement

This work was supported by World Premier International Research Center Initiative (WPI), MEXT, Japan and by the Ministry of Education and Science of the Russian Federation in the framework of Increase Competitiveness Program of NUST«MISiS» № К1-2015-026. This work was also supported by Japan Society for Promotion of Science (JSPS) Grant-in-aid for Scientific Research (KAKENHI) (Grant Number #16K18244).

### Competing interest statement

The authors declare no conflict of interest.

### Additional information

No additional information is available for this paper.
